# Cancer incidence in the Somali population of Olmsted County: A Rochester epidemiology project study

**DOI:** 10.1002/cam4.6558

**Published:** 2023-09-23

**Authors:** Ahmed A. Mohamed, Alanna M. Chamberlain, Kathleen J. Yost, Gregory Jenkins, Lila J. Finney Rutten, Mark L. Wieland, Jane W. Njeru

**Affiliations:** ^1^ Department of Medicine Mayo Clinic Rochester Minnesota USA; ^2^ Department of Quantitative Health Sciences Mayo Clinic Rochester Minnesota USA; ^3^ Department of Cardiovascular Medicine Mayo Clinic Rochester Minnesota USA

**Keywords:** epidemiology and prevention, liver cancer, neoplasm, risk factors

## Abstract

**Background:**

Somali immigrants and refugees constitute one of the largest African ethnic groups immigrating to the United States over the past three decades with the majority resettling in the state of Minnesota. Previous studies have documented significant cancer screening disparities between the Somali population and the general population. However, little is known about cancer incidence among Somali groups living in the United States.

**Methods:**

We determined the incidence of 18 types or sites of malignancy using ICD‐9 and ICD‐10 codes and compared them between Somali and non‐Somali populations in Olmsted County, Minnesota utilizing the Rochester Epidemiology Project medical records‐linkage infrastructure for the years 2000–2020. Poisson regression models were used to model the rates for each malignancy.

**Results:**

There was a higher incidence and relative risk of liver malignancies among the Somali population versus non‐Somali population, but lower relative risk and incidence of the following malignancies: breast, cervical, and melanoma. After direct age‐sex adjustment to the United States 2000 Census population, liver was the most common cancer in Somali men, while breast cancer was the most common malignancy in women.

**Conclusion:**

Malignancies related to infectious agents such as viral hepatitis have a higher incidence in the Somali immigrant population of Olmsted County. There is a lower incidence of malignancies related to lifestyle factors in this Somali population. Findings of this study may help inform cancer prevention and screening strategies among Somali communities in the United States.

## 1 INTRODUCTION

People from Somalia constitute one of the largest groups of African immigrants and refugees resettling in the United States since Civil War erupted in the 1990s in Somalia. The majority of Somali refugees to the United States have relocated to the state of Minnesota. There are significant known disparities in cancer screening among Somali patients compared to the general population.^1^, ^2^ However, little is known about the cancer incidence in the Somali diaspora and whether there is a disparity in the incidence of screenable malignancies. Determining cancer incidence will help in the development of targeted interventions to reduce cancer morbidity and mortality.

In the single United States study to examine cancer incidence among immigrants from sub‐Saharan Africa, Medhanie et al. found a higher incidence of infection‐related cancers (liver, stomach, and Kaposi sarcoma), blood cancers (leukemia and non‐Hodgkin lymphoma), prostate cancer, and thyroid cancers (females only) in African‐born blacks as compared to US‐born non‐Hispanic blacks.^3^ However, cancer incidence was not reported by country of birth though Somalia was included in the list of countries in the Eastern Africa region. The results may have also been skewed by African countries with larger immigrant populations in the United States.

There have been two studies on cancer incidence conducted in Somalia. Both were at healthcare institutions in the capital city of Mogadishu. Esophageal cancer was the most common malignancy identified in both studies, comprising 32.3% of reported malignancies in Bas et al^4^ and 21.7% in Tahtabasi et al.^5^ Non‐Hodgkin lymphoma (8.7%) and hepatocellular carcinoma (6.5%) were the second and third most common malignancies in Bas et al., while hepatocellular carcinoma (7.6%) and breast cancer (7.3%) were second and third in Tahtabasi et al. There are no formal cancer registries in Somalia.^6^

A challenge of conducting population‐based research among Somali groups in the U.S. is that registry data and national surveys do not contain the necessary granularity to distinguish Somali participants from other black or African American populations. Therefore, the objective of this study was to determine population‐based cancer incidence for Somali residents in Olmsted County Minnesota, where Somali individuals and families have been continuously re‐settled since the early 1990s. We accomplished this using the Rochester Epidemiology Project (REP) infrastructure, which contains longitudinal health information on nearly all residents living in this region.^7^, ^8^, ^9^

## 2 METHODS

### 2.1 Data source

The Rochester Epidemiology Project is a medical records‐linkage system for people living in southern Minnesota.^8^ It was established in 1966 and has been continuously updated with data from multiple institutions providing care for patients in this region.^7^, ^8^ The REP includes demographic information, medical identification numbers, and diagnostic codes for medical conditions and surgical procedures.^9^ The REP population was found to be generalizable to the population of the state of Minnesota and the Upper Midwest though less diverse and wealthier than the general United States population from 1970 to 2000.^10^ However, studies on cancer incidence in Olmsted County have shown similar trends as the rest of the United States population.^11^, ^12^

We determined the incidence of the most common cancer diagnoses and compared them between Somali and non‐Somali populations during the years 2000–2020 in Olmsted County. The list of diagnoses was determined by selecting the 10 malignancies with the highest incidence in each of the two studies conducted in Somalia,^4^, ^5^ one study in the United States on Sub‐Saharan African‐born Blacks,^3^ a study on cancer incidence in Sub‐Saharan African countries based on the GLOBOCON database,^13^ and the SEER 5‐year age‐adjusted incidence rates from 2015 to 2019. In total, 18 cancer sites or diagnoses were included in this study.^14^

A person was identified as Somali if one or more of their medical records indicated that Somalia was their country of origin, that they were Somali ethnicity, that they speak Somali, or if the text string ‘Somali’ was found in a visit note.^2^

We electronically searched REP indexes to extract demographic variables and diagnostic codes based on the *International Classification of Diseases*, *Ninth Revision* (*ICD*‐*9*), and *International Classification of Diseases*, *Tenth Revision* (*ICD*‐*10*).^15^ ICD‐9 and ICD‐10 codes excluded prior malignancies (i.e., history of a malignancy) outside of the study period. Diagnosis codes with the description “in remission” or “relapse” were excluded for hematologic malignancies. Code sets are included in the Table S1.

This study was approved by the Mayo Clinic and Olmsted Medical Center Institutional Review Boards. The study was considered minimal risk; therefore, the requirement for informed consent was waived. However, records of any patient who had not provided authorization for their medical records to be used for research, as per Minnesota statute 144.335, were not reviewed.

### 2.2 Statistical analysis

The annual incidence rate of a particular malignancy was calculated as the number of individuals with a new malignancy divided by the REP census population (i.e., after excluding individuals with a history of the same malignancy type). Poisson regression was used to model the rate of a particular malignancy. The following variables were included in the Poisson model: year, age, sex, and Somali descent. Relative risks for malignancy comparing Somali to the non‐Somali Olmsted County population were calculated from the Poisson regression models. Wald tests were used to test for malignancy risk differences associated with being in the Somali population. A conservative Bonferroni multiple testing correction was used to assess statistical significance correcting for 18 tests, by setting the threshold at *p*‐value <0.0028 (0.05/18). Malignancy rates were also computed over 2000–2020 using new cases of malignancy as the numerator and person‐years as the denominator. These rates were directly adjusted separately in the Somali and non‐Somali populations to the 2000 United States Census total population, adjusting for age and sex; and reported per 100,000 individuals.^16^

## 3 RESULTS

The Somali population was consistently about 10 years younger than the non‐Somali population in Olmsted County between 2000 and 2020, with a mean (SD) age of 24 (17) versus 35 (22) years in 2000; 27 (20) versus 37 (23) years in 2010; and 29 (20) versus 39 (23) years in 2020. However, the Somali population was similar in terms of proportions of males and females; with females making up 50%, 54%, and 52% in the Somali population for 2000, 2010, and 2020 compared to 52%, 52%, and 53% in the non‐Somali population (Table 1).

**TABLE 1:** Age and sex distribution of Olmsted county Somali and non‐Somali populations for select years, 2000–2020.

	2000		2010		2020	
	Somali (*n* = 1409)	Non‐Somali (*n* = 126,026)	Somali (*n* = 2705)	Non‐Somali (*n* = 142,579)	Somali (*n* = 3878)	Non‐Somali (*n* = 150,101)
Age, years						
0–19	625 (44.4)	37,046 (29.4)	1120 (41.4)	38,893 (27.3)	1494 (38.5)	37,029 (24.7)
20–39	542 (38.5)	39,332 (31.2)	930 (34.4)	40,995 (28.8)	1321 (34.1)	44,449 (29.6)
40–59	166 (11.8)	31,999 (25.4)	430 (15.9)	38,357 (26.9)	727 (18.7)	35,122 (23.4)
60–79	74 (5.3)	13,791 (10.9)	195 (7.2)	19,126 (13.4)	277 (7.1)	27,098 (18.1)
≥80	2 (0.1)	3855 (3.1)	30 (1.1)	5208 (3.7)	59 (1.5)	6403 (4.3)
Sex						
Female	705 (50.0)	65,021 (51.6)	1452 (53.7)	74,374 (52.2)	2013 (51.9)	79,015 (52.6)
Male	704 (50.0)	61,005 (48.4)	1253 (46.3)	68,205 (47.8)	1865 (48.1)	71,086 (47.4)

*Note*: *N* (%) for particular year and group.

**FIGURE 1:**
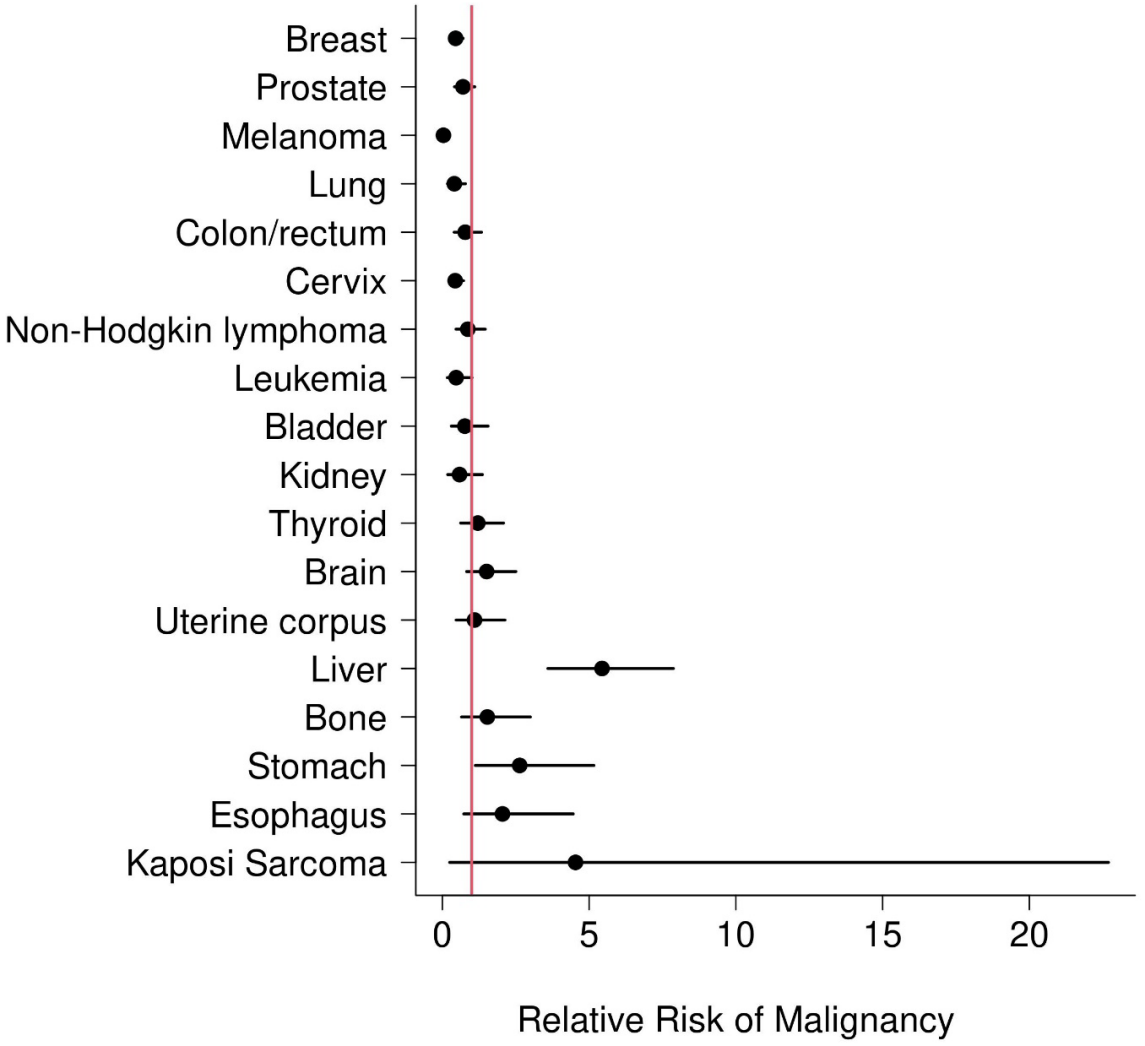
Age, Sex and Year‐Adjusted Relative Risk of Malignancy Somali vs. Non−Somali (2000−2020).

Of the 18 types or sites of malignancies reviewed between 2000 and 2020, annual incidence of 10 of the malignancies trended lower in the Somali population (Figure 1; Table 2). However, with a conservative Bonferroni multiple testing correction, only melanoma ((RR) (95% CI): (0.04 (0.00, 0.16)), breast (0.45 (0.26, 0.71)), and cervical cancer (0.44 (0.24, 0.72)) were found to be statistically significant. The Somali population was found to be at greater risk of liver and intrahepatic bile duct cancers (RR (95% CI)): (5.44 (3.60, 7.87)).

**TABLE 2:** Relative risk of malignancy for Somali versus non‐Somali and direct age‐ and sex‐adjusted rates (2000 United States Census total population).

Malignancy	RR (95% CI)	*p*‐value^b^	Rate in Somali population^a^	Rate in non‐Somali population^a^
Breast	0.45 (0.26, 0.71)	**0**.**0003**	74.49 (36.51, 112.47)	188.72 (181.82, 195.62)
Prostate	0.70 (0.41, 1.10)	0.1323	122.00 (61.10, 182.91)	187.01 (179.35, 194.68)
Melanoma	0.04 (0.00, 0.16)	<**0**.**0001**	1.06 (−1.02, 3.13)	70.59 (67.58, 73.61)
Lung	0.41 (0.17, 0.79)	0.0053	17.35 (3.89, 30.81)	59.84 (57.1, 62.59)
Colon/rectum	0.78 (0.40, 1.34)	0.3866	48.34 (10.48, 86.20)	47.43 (44.97, 49.89)
Cervix	0.44 (0.24, 0.72)	**0**.**0006**	50.09 (21.14, 79.04)	89.02 (84.28, 93.76)
Non‐Hodgkin lymphoma	0.87 (0.46, 1.46)	0.6165	39.70 (15.17, 64.23)	39.63 (37.39, 41.88)
Leukemia	0.47 (0.17, 1.01)	0.0540	14.15 (0.99, 27.31)	30.70 (28.73, 32.66)
Bladder	0.77 (0.30, 1.56)	0.4960	19.22 (3.78, 34.66)	27.67 (25.82, 29.52)
Kidney	0.59 (0.18, 1.37)	0.2417	10.85 (−0.21, 21.92)	21.22 (19.59,22.86)
Thyroid	1.21 (0.62, 2.08)	0.5493	29.24 (10.90, 47.58)	20.23 (18.61, 21.84)
Brain	1.51 (0.82, 2.51)	0.1703	26.83 (10.99, 42.67)	19.83 (18.23, 21.42)
Uterine corpus	1.10 (0.47, 2.13)	0.8132	36.84 (8.61, 65.06)	34.29 (31.46, 37.12)
Liver	5.44 (3.60, 7.87)	<**0**.**0001**	94.32 (56.62, 132.02)	14.92 (13.56, 16.29)
Bone	1.53 (0.65, 3.00)	0.2972	20.72 (2.68, 38.76)	11.44 (10.24, 12.65)
Stomach	2.63 (1.12, 5.16)	0.0285	21.87 (5.22, 38.51)	8.88 (7.84, 9.93)
Esophagus	2.05 (0.73, 4.46)	0.1549	15.37 (1.74, 28.99)	7.95 (6.96, 8.93)
Kaposi Sarcoma	4.54 (0.25, 22.70)	0.2335	1.49 (−1.43, 4.42)	0.53 (0.27, 0.80)

^a^ Age‐adjusted (for prostate and cervix) or sex‐ and age‐adjusted (for the remaining malignancies) malignancy rate per 100,000 (95% CI) adjusted to the 2000 United States census total population.

^b^ Bonferroni corrected *p*‐value was 0.0028. *p*‐values in bold are statistically significant after correction.

## 4 DISCUSSION

This population‐based, retrospective cohort study of Somali residents in Olmsted County, Minnesota demonstrated statistically significant elevated risk for liver cancer compared to the non‐Somali population, and a lower risk of melanoma, cervical, and breast cancers after adjusting for multiple comparisons. In general, this study reflects a higher risk of cancers that are mechanistically linked to infectious causes, and a lower risk of cancers linked to health behaviors (smoking, low physical activity, low dietary quality) and lifestyle choice (parity, breastfeeding).

These findings are consistent with the study by Medhanie et al on cancer incidence among Sub‐Saharan African immigrants to the U.S.^3^ Furthermore, Parkin et al, reported that 28.7% of cancers in Sub‐Saharan Africa are due to infectious agents.^17^ Among the Somali population, there is a high prevalence of viral hepatitis in the U.S. and abroad.^18^, ^19^, ^20^, ^21^, ^22^ Alcohol consumption is low in the Somali population,^23^, ^24^ and metabolic syndrome is lower in recent immigrants and in Somalia.^25^, ^26^ This emphasizes the importance of clinic and community‐based campaigns to promote screening and treatment for hepatitis B and C, which contribute to hepatobiliary cancers. Similarly, the high risk of gastric cancer among Somali residents (RR (95% CI): 2.63 (1.12, 5.16)), although not statistically significantly higher than for non‐Somalis, partially reflects the causative links between *Helicobacter pylori* (*H*. *pylori*) infection and gastric cancer.^27^ Current guidelines do not endorse universal *H*. *pylori* screening for refugees to the United States, but this study suggests that this preventive approach should be considered for Somali patients. There was a trend toward a higher risk of Kaposi sarcoma, which is linked to HIV infection, though this was not statistically significant (RR (95% CI): 4.54 (0.25, 22.7)).

One exception to the higher risk for infection‐related malignancies is cervical cancer. The Somali population in this study had a lower incidence of cervical cancer, a screenable malignancy associated with HPV infection. Recent estimates show that cervical cancer is the second most common malignancy among women and third most common overall in both sexes combined in Sub‐Saharan Africa though Somalia is not part of any registry.^6^, ^13^ Still, cervical cancer is the fourth most common malignancy in this study and third most common among women behind liver and breast cancer. Cultural and religious beliefs among Somalis (e.g., prohibition of premarital sex and multiple sexual partners) may contribute to reduced risk of HPV infection and, consequently, lower incidence of cervical cancer. Cultural and religious beliefs in this population is associated with lower perceived risk of HPV infection.^28^, ^29^, ^30^ As a result, HPV vaccination is lower in Somali adolescents as compared to non‐Hispanic white adolescents.^31^ Cervical cancer screening is also lower among Somali adults.^2^, ^30^, ^32^, ^33^

We found a lower risk of cancers that are primarily linked to lifestyle factors among Somali patients compared with non‐Somali patients. In 2009, smoking prevalence was 20.6% among US adults (23.5% male, 17.9% women).^34^ However, smoking prevalence among Somali adults in Minnesota was 24% (44% male, 4% female) in a 2012 study based on a survey conducted in 2009.^35^ Somali youth reported a higher prevalence of hookah use (5.0% vs. 2.4%, *p* < 0.01) as compared to non‐Somali peers in Minnesota but lower use of other tobacco products.^36^ Tobacco use is associated with multiple malignancies including gastric cancers, which has a higher incidence in this Somali population.^37^ Similarly, hookah use is also associated with multiple malignancies, especially cancers of the head and neck, lung, and esophagus.^38^ Programs for tobacco use prevention and cessation will help to further reduce the risk of cancer in this population.

In a past study of Somali residents in Olmsted County, physical activity and dietary quality were found to be relatively high.^39^ However, acculturation has been shown to result in worsening diet, physical inactivity, and increasing body mass index among immigrants in high‐income countries.^25^, ^26^ Previous research has shown that interventions in the first 10 years after resettlement may be particularly powerful in preventing worsening diet and physical activity after immigration.^25^ Obesity is associated with an increased risk of multiple cancers including at the following sites: esophagus, gastric cardia, colon and rectum, liver, gallbladder, pancreas, breast, uterus, ovary, kidney, meningioma, thyroid.^40^

Screening for breast, cervical, and colorectal cancer has been found to be lower among Somali populations as compared to non‐Somalis in Minnesota and Maine in the United States.^1^, ^2^ Colorectal cancer screening is found to be 35.5% among the Somali population of Minnesota versus 73.5% for the overall population.^41^ Cervical cancer screening has also been found to be low among Somali women in Washington State.^42^ In Europe, Somali immigrants in Finland had an age‐adjusted cervical cancer screening rate of 41% as compared with 94% among non‐Somali Finns.^33^ Similar disparities have been noted in studies conducted in Denmark and Norway.^32^, ^43^

Despite these screening disparities, our study found lower incidence rates of these cancers among Somali patients compared with non‐Somali patients. If Somali populations follow similar health behavior trends to other immigrant groups to the U.S. (e.g., decline in physical activity and dietary quality), then screening interventions that target Somali patients will become increasingly important. To date, several studies have explored interventions to improve cancer screening disparities for breast, cervical and/or colorectal cancer in the Somali immigrant population and have shown promising results.^30^, ^44^, ^45^, ^46^, ^47^, ^48^, ^49^, ^50^ This study suggests that cancer screening for new Somali immigrants should especially focus on infection‐related cancers through hepatitis B and C testing, HPV testing, and testing for *H*. *pylori* infection. Future studies on larger Somali populations should be conducted to confirm these findings and to longitudinally study the effect of acculturation constructs on cancer incidence over time.

### 4.1 Limitations

Our inclusion criteria may have missed patients whose medical records did not identify them as Somali or as requiring a Somali interpreter. We considered including patients with common Somali surnames but determined that only 50% of those patients were Somali after reviewing a sample of 50 charts. We also reviewed a random sample of 50 patients identified as Somali according to our study methods and confirmed that the list was 100% accurate. We also reviewed a random list of patients that were excluded who were not identified as Somali or did not have common Somali surnames. We confirmed that this list did not have any Somali patients. From this chart review, we estimate that we are missing approximately 25% of the total Somali population in Olmsted County with our method of patient identification. Nevertheless, strengths of our study include a geographically enumerated population and the comprehensive capture of medical record information through the Rochester Epidemiology Project, from which to draw population‐based conclusions. There is potential for selection bias since the Somali participants who did not provide research authorization were excluded. However, only 2% of Olmsted County residents have refused to allow their medical records to be used for research since the year 2000.^9^

### 4.2 Conclusion

Malignancies related to infectious agents such as viral hepatitis have a higher incidence in the Somali immigrant population of Olmsted County. Screening for and treating infectious agents can help reduce the incidence of associated cancers in the Somali population. There is a lower incidence of malignancies associated with lifestyle factors in the Somali population. Future studies on acculturation and cancer incidence in this population are needed to follow up on these results.

## AUTHOR CONTRIBUTIONS

**Ahmed A. Mohamed:** Conceptualization (equal); funding acquisition (lead); investigation (lead); methodology (lead); writing – original draft (lead); writing – review and editing (equal). **Alanna M. Chamberlain:** Formal analysis (supporting); investigation (equal); methodology (supporting); supervision (equal); writing – review and editing (equal). **Kathleen J. Yost:** Investigation (equal); writing – review and editing (equal). **Gregory Jenkins:** Data curation (equal); formal analysis (lead); writing – review and editing (equal). **Lila J Finney Rutten:** Investigation (equal); writing – review and editing (equal). **Mark L. Wieland:** Conceptualization (equal); investigation (equal); supervision (supporting); writing – review and editing (equal). **Jane W. Njeru:** Conceptualization (equal); investigation (equal); supervision (supporting); writing – review and editing (equal).

## FUNDING INFORMATION

Funding was provided by Primary Care in Southeast Minnesota in conjunction with the Population Health Science Scholars Program in the Mayo Clinic Robert D. and Patricia E. Kern Center.

## CONFLICT OF INTEREST STATEMENT

To the best of our knowledge, no relevant conflict of interest, financial or other, exists.

## Supporting information


**Table S1.**: Click here for additional data file.

## Data Availability

The data that support the findings of this study are available from the corresponding author upon reasonable request.
