# Supplementary material for: Cancer incidence in the Somali population of Olmsted County: A Rochester epidemiology project study
Source: Cancer Med. 2023 Sep 23;12(19):20027–34. doi: 10.1002/cam4.6558 (PMC10587927; doi:10.1002/cam4.6558)
Supplement: Supplementary file 1 — Table S1.: [file CAM4-12-20027-s001.docx]

|  | **Incident Cancer ICD Codes** | | **Exclusion ICD Codes (codes in Incident columns + these extra code)** | |
| --- | --- | --- | --- | --- |
| **Malignancy (Diagnosis or Site)** | **ICD-10 code** | **ICD-9 code** | **ICD-10 code** | **ICD-9 code** |
| Bladder | C67.0, C67.1, C67.2, C67.3, C67.4, C67.5, C67.6, C67.7, C67.8, C67.9, D09.0 | 188, 188.0, 188.1, 188.2, 188.3, 188.4, 188.5, 188.6, 188.7, 188.8, 188.9, 233.7 | Z85.51  (personal history codes start with Z) | V10.51  (personal history codes start with V) |
| Breast | C50.011, C50.012, C50.019, C50.021, C50.022, C50.029, C50.111, C50.112, C50.119, C50.121, C50.122, C50.129, C50.211, C50.212, C50.219, C50.221, C50.222, C50.229, C50.311, C50.312, C50.319, C50.321, C50.322, C50.329, C50.411, C50.412, C50.419, C50.421, C50.422, C50.429, C50.511, C50.512, C50.519, C50.521, C50.522, C50.529, C50.611, C50.612, C50.619, C50.621, C50.622, C50.629, C50.811, C50.812, C50.819, C50.821, C50.822, C50.829, C50.911, C50.912, C50.919, C50.921, C50.922, C50.929, D05.00, D05.01, D05.02, D05.10, D05.11, D05.12, D05.80, D05.81, D05.82, D05.90, D05.91, D05.92 | 174, 174.0, 174.1, 174.2, 174.3, 174.4, 174.5, 174.6, 174.8, 174.9, 175, 175.0, 175.9, 233.0 | Z85.3 | V10.3 |
| Cervix | C53.0, C53.1, C53.8, C53.9, D06.0, D06.1, D06.7, D06.9 | 180, 180.0, 180.1, 180.8, 180.9, 233.1 | Z85.41 | V10.41 |
| Colon/rectum | C18.0, C18.1, C18.2, C18.3, C18.4, C18.5, C18.6, C18.7, C18.8, C18.9, C19, C20, C21.8, D01.0, D01.1, D01.2 | 153, 153.0, 153.1, 153.2, 153.3, 153.4, 153.5, 153.6, 153.7, 153.8, 153.9, 154, 154.0, 154.1, 154.8, 230.3, 230.4 | Z85.030, Z85.038, Z85.040, Z85.048  Wrong site or type of malignancy:  C21.0, C21.1, C21.2, D01.3, D01.40, D01.49 | V10.05, V10.06  Wrong site or type of malignancy:  154.2, 154.3, 230.5, 230.6, 230.7 |
| Esophagus | C15.3, C15.4, C15.5, C15.8, C15.9, D00.1 | 150, 150.0, 150.1, 150.2, 150.3, 150.4, 150.5, 150.8, 150.9, 230.1 | Z85.01 | V10.03 |
| Kidney | C64.1, C64.2, C64.9 | 189, 189.0 | Z85.520, Z85.528  Wrong site or type of malignancy:  D09.19 | V10.52  Wrong site or type of malignancy:  233.9 |
| Liver | C22.0, C22.1, C22.2, C22.3, C22.4, C22.7, C22.8, C22.9 | 155, 155.0, 155.1, 155.2 | Z85.05  Wrong site or type of malignancy:  D01.5 | V10.07, V10.09  Wrong site or type of malignancy:  230.8 |
| Lung | C34.00, C34.01, C34.02, C34.10, C34.11, C34.12, C34.2, C34.30, C34.31, C34.32, C34.80, C34.81, C34.82, C34.90, C34.91, C34.92 | 162, 162.0, 162.2, 162.3, 162.4, 162.5, 162.8, 162.9 | Z85.110, Z85.118, Z85.12  Wrong site or type of malignancy:  C33, C38.4, C38.8, C39.0, C39.9 | V10.1, V10.11, V10.12  Wrong site or type of malignancy:  162, 162.0, 163, 163.0, 163.1, 163.8, 163.9 |
| Melanoma | C43.0, C43.10, C43.11, C43.111, C43.112, C43.12, C43.121, C43.122, C43.20, C43.21, C43.22, C43.30, C43.31, C43.39, C43.4, C43.51, C43.52, C43.59, C43.60, C43.61, C43.62, C43.70, C43.71, C43.72, C43.8, C43.9, D03.0, D03.10, D03.11, D03.111, D03.112, D03.12, D03.121, D03.122, D03.20, D03.21, D03.22, D03.30, D03.39, D03.4, D03.51, D03.52, D03.59, D03.60, D03.61, D03.62, D03.70, D03.71, D03.72, D03.8, D03.9 | 172, 172.0, 172.1, 172.2, 172.3, 172.4, 172.5, 172.6, 172.7, 172.8, 172.9 | Z85.820, Z86.006  Wrong site or type of malignancy:  C51.0, C51.1, C51.2, C51.8, C51.9, C52, C69.00, C69.01, C69.02, C69.10, C69.11, C69.12, C69.20, C69.21, C69.22, C69.30, C69.31, C69.32, C69.40, C69.41, C69.42, C69.50, C69.51, C69.52, C69.60, C69.61, C69.62, C69.80, C69.81, C69.82, C69.90, C69.91, C69.92 | V10.82  Wrong site or type of malignancy:  184.0, 184.1, 184.2, 184.3, 184.4, 184.8, 184.9, 187.1, 187.2, 187.3, 187.4, 187.5, 187.6, 187.7, 187.8, 187.9, 190, 190.0, 190.1, 190.2, 190.3, 190.4, 190.5, 190.6, 190.7, 190.8, 190.9 |
| Prostate | C61, D07.5 | 185, 233.4 | Z85.46, Z86.002 | V10.46 |
| Stomach | C16.0, C16.1, C16.2, C16.3, C16.4, C16.5, C16.6, C16.8, C16.9, D00.2 | 151, 151.0, 151.1, 151.2, 151.3, 151.4, 151.5, 151.6, 151.8, 151.9, 230.2 | Z85.020, Z85.028 | V10.04 |
| Thyroid | C73, D09.3 | 193, 234.8 | Z85.850 | V10.87 |
| Uterine corpus | C54.0, C54.1, C54.2, C54.3, C54.8, C54.9, C55, D07.0 | 179, 182, 182.0, 182.1, 182.8, 233.2 | Z85.42 | V10.42 |
| Brain | C71.0, C71.1, C71.2, C71.3, C71.4, C71.5, C71.6, C71.7, C71.8, C71.9, C70.0, C72.20, C72.21, C72.22, C72.30, C72.31, C72.32, C72.40, C72.41, C72.42, C72.50, C72.59, C75.1, C75.2, C75.3 | 191, 191.0, 191.1, 191.2, 191.3, 191.4, 191.5, 191.6, 191.7, 191.8, 191.9, 192, 192.0, 192.1, 194.3, 194.4 | Z85.841 | V10.85 |
| Bone | C40.00, C40.01, C40.02, C40.10, C40.11, C40.12, C40.20, C40.21, C40.22, C40.30, C40.31, C40.32, C40.80, C40.81, C40.82, C40.90, C40.91, C40.92, C41.0, C41.1, C41.2, C41.3, C41.4, C41.9 | 170, 170.0, 170.1, 170.2, 170.3, 170.4, 170.5, 170.6, 170.7, 170.8, 170.9 | Z85.830  Wrong site or type of malignancy:  C79.51, C79.52 | V10.81  Wrong site or type of malignancy:  198.5 |
| Kaposi Sarcoma | C46.0, C46.1, C46.2, C46.3, C46.4, C46.50, C46.51, C46.52, C46.7, C46.9 | 176, 176.0, 176.1, 176.2, 176.3, 176.4, 176.5, 176.8, 176.9 | N/A | N/A |
| Leukemia | C90.10, C91.00, C91.10, C91.30, C91.40, C91.50, C91.60, C91.A0, C91.Z0, C91.90, C92.00, C92.10, C92.20, C92.30, C92.40, C92.50, C92.60, C92.A0, C92.Z0, C92.90, C93.00, C93.10, C93.30, C93.Z0, C93.90, C94.00, C94.20, C94.30, C94.40, C94.6, C94.80, C95.00, C95.10, C95.90 | 202.4, 202.40, 202.41, 202.42, 202.43, 202.44, 202.45, 202.46, 202.47, 202.48, 203.1, 203.10, 203.8, 203.80, 204, 204.0, 204.00, 204.1, 204.10, 204.2, 204.20, 204.8, 204.80, 204.9, 204.90, 205, 205.0, 205.00, 205.1, 205.10, 205.2, 205.20, 205.3, 205.30, 205.8, 205.80, 205.9, 205.90, 205.92, 206, 206.0, 206.00, 206.1, 206.10, 206.2, 206.20, 206.8, 206.80, 206.9, 206.90, 207, 207.0, 207.00, 207.1, 207.10, 207.2, 207.20, 207.8, 207.80, 208, 208.0, 208.00, 208.1, 208.10, 208.2, 208.20, 208.8, 208.80, 208.9, 208.90 | Z85.6  Relapse or remission codes:  C90.11, C90.12, C91.01, C91.02, C91.11, C91.12, C91.31, C91.32, C91.41, C91.42, C91.51, C91.52, C91.61, C91.62, C91.A1, C91.A2, C91.Z1, C91.Z2, C91.91, C91.92, C92.01, C92.02, C92.11, C92.12, C92.21, C92.22, C92.31, C92.32, C92.41, C92.42, C92.51, C92.52, C92.61, C92.62, C92.A1, C92.A2, C92.Z1, C92.Z2, C92.91, C92.92, C93.01, C93.02, C93.11, C93.12, C93.31, C93.32, C93.Z1, C93.Z2, C93.91, C93.92, C94.01, C94.02, C94.21, C94.22, C94.31, C94.32, C94.41, C94.42,  C94.81, C94.82, C95.01, C95.02, C95.11, C95.12, C95.91, C95.92 | V10.6, V10.60, V10.61, V10.62, V10.63, V10.69  Relapse or remission codes:  203.11, 203.12, 203.81, 203.82, 204.01, 204.02,  204.11, 204.12, 204.21, 204.22, 204.81, 204.82,  204.91, 204.92, 205.01, 205.02, 205.11, 205.12,  205.21, 205.22, 205.31, 205.32, 205.81, 205.82,  205.91, 206.01, 206.02,  206.11, 206.12, 206.21, 206.22, 206.81, 206.82,  206.91, 206.92, 207.01, 207.02, 207.11, 207.12,  207.21, 207.22, 207.81, 207.82, 208.01, 208.02,  208.11, 208.12, 208.21, 208.22, 208.81, 208.82,  208.91, 208.92 |
| Non-Hodgkin lymphoma | C82.00, C82.01, C82.02, C82.03, C82.04, C82.05, C82.06, C82.07, C82.08, C82.09, C82.10, C82.11, C82.12, C82.13, C82.14, C82.15, C82.16, C82.17, C82.18, C82.19, C82.20, C82.21, C82.22, C82.23, C82.24, C82.25, C82.26, C82.27, C82.28, C82.29, C82.30, C82.31, C82.32, C82.33, C82.34, C82.35, C82.36, C82.37, C82.38, C82.39, C82.40, C82.41, C82.42, C82.43, C82.44, C82.45, C82.46, C82.47, C82.48, C82.49, C82.50, C82.51, C82.52, C82.53, C82.54, C82.55, C82.56, C82.57, C82.58, C82.59, C82.60, C82.61, C82.62, C82.63, C82.64, C82.65, C82.66, C82.67, C82.68, C82.69, C82.80, C82.81, C82.82, C82.83, C82.84, C82.85, C82.86, C82.87, C82.88, C82.89, C82.90, C82.91, C82.92, C82.93, C82.94, C82.95, C82.96, C82.97, C82.98, C82.99, C83.00, C83.01, C83.02, C83.03, C83.04, C83.05, C83.06, C83.07, C83.08, C83.09, C83.10, C83.11, C83.12, C83.13, C83.14, C83.15, C83.16, C83.17, C83.18, C83.19, C83.30, C83.31, C83.32, C83.33, C83.34, C83.35, C83.36, C83.37, C83.38, C83.39, C83.50, C83.51, C83.52, C83.53, C83.54, C83.55, C83.56, C83.57, C83.58, C83.59, C83.70, C83.71, C83.72, C83.73, C83.74, C83.75, C83.76, C83.77, C83.78, C83.79, C83.80, C83.81, C83.82, C83.83, C83.84, C83.85, C83.86, C83.87, C83.88, C83.89, C83.90, C83.91, C83.92, C83.93, C83.94, C83.95, C83.96, C83.97, C83.98, C83.99, C84.00, C84.01, C84.02, C84.03, C84.04, C84.05, C84.06, C84.07, C84.08, C84.09, C84.10, C84.11, C84.12, C84.13, C84.14, C84.15, C84.16, C84.17, C84.18, C84.19, C84.40, C84.41, C84.42, C84.43, C84.44, C84.45, C84.46, C84.47, C84.48, C84.49, C84.60, C84.61, C84.62, C84.63, C84.64, C84.65, C84.66, C84.67, C84.68, C84.69, C84.70, C84.71, C84.72, C84.73, C84.74, C84.75, C84.76, C84.77, C84.78, C84.79, C84.7A, C84.A0, C84.A1, C84.A2, C84.A3, C84.A4, C84.A5, C84.A6, C84.A7, C84.A8, C84.A9, C84.Z0, C84.Z1, C84.Z2, C84.Z3, C84.Z4, C84.Z5, C84.Z6, C84.Z7, C84.Z8, C84.Z9, C84.90, C84.91, C84.92, C84.93, C84.94, C84.95, C84.96, C84.97, C84.98, C84.99, C85.10, C85.11, C85.12, C85.13, C85.14, C85.15, C85.16, C85.17, C85.18, C85.19, C85.20, C85.21, C85.22, C85.23, C85.24, C85.25, C85.26, C85.27, C85.28, C85.29, C85.80, C85.81, C85.82, C85.83, C85.84, C85.85, C85.86, C85.87, C85.88, C85.89, C85.90, C85.91, C85.92, C85.93, C85.94, C85.95, C85.96, C85.97, C85.98, C85.99, C86.0, C86.1, C86.2, C86.3, C86.4, C86.5, C86.6, C88.0, C88.2, C88.3, C88.4, C88.8, C88.9 | 200, 200.0, 200.00, 200.01, 200.02, 200.03, 200.04, 200.05, 200.06, 200.07, 200.08, 200.1, 200.10, 200.11, 200.12, 200.13, 200.14, 200.15, 200.16, 200.17, 200.18, 200.2, 200.20, 200.21, 200.22, 200.23, 200.24, 200.25, 200.26, 200.27, 200.28, 200.3, 200.30, 200.31, 200.32, 200.33, 200.34, 200.35, 200.36, 200.37, 200.38, 200.4, 200.40, 200.41, 200.42, 200.43, 200.44, 200.45, 200.46, 200.47, 200.48, 200.5, 200.50, 200.51, 200.52, 200.53, 200.54, 200.55, 200.56, 200.57, 200.58, 200.6, 200.60, 200.61, 200.62, 200.63, 200.64, 200.65, 200.66, 200.67, 200.68, 200.7, 200.70, 200.71, 200.72, 200.73, 200.74, 200.75, 200.76, 200.77, 200.78, 200.8, 200.80, 200.81, 200.82, 200.83, 200.84, 200.85, 200.86, 200.87, 200.88, 202, 202.0, 202.00, 202.01, 202.02, 202.03, 202.04, 202.05, 202.06, 202.07, 202.08, 202.1, 202.10, 202.11, 202.12, 202.13, 202.14, 202.15, 202.16, 202.17, 202.18, 202.2, 202.20, 202.21, 202.22, 202.23, 202.24, 202.25, 202.26, 202.27, 202.28, 202.3, 202.30, 202.31, 202.32, 202.33, 202.34, 202.35, 202.36, 202.37, 202.38, 202.4, 202.40, 202.41, 202.42, 202.43, 202.44, 202.45, 202.46, 202.47, 202.48, 202.5, 202.50, 202.51, 202.52, 202.53, 202.54, 202.55, 202.56, 202.57, 202.58, 202.6, 202.60, 202.61, 202.62, 202.63, 202.64, 202.65, 202.66, 202.67, 202.68, 202.7, 202.70, 202.71, 202.72, 202.73, 202.74, 202.75, 202.76, 202.77, 202.78, 202.8, 202.80, 202.81, 202.82, 202.83, 202.84, 202.85, 202.86, 202.87, 202.88 | Z85.71, Z85.72  Hodgkin lymphoma codes:  C81.00, C81.01, C81.02, C81.03, C81.04, C81.05, C81.06, C81.07, C81.08, C81.09, C81.10, C81.11, C81.12, C81.13, C81.14, C81.15, C81.16, C81.17, C81.18, C81.19, C81.20, C81.21, C81.22, C81.23, C81.24, C81.25, C81.26, C81.27, C81.28, C81.29, C81.30, C81.31, C81.32, C81.33, C81.34, C81.35, C81.36, C81.37, C81.38, C81.39, C81.40, C81.41, C81.42, C81.43, C81.44, C81.45, C81.46, C81.47, C81.48, C81.49, C81.70, C81.71, C81.72, C81.73, C81.74, C81.75, C81.76, C81.77, C81.78, C81.79, C81.90, C81.91, C81.92, C81.93, C81.94, C81.95, C81.96, C81.97, C81.98, C81.99 | V10.7, V10.71, V10.72, V10.79  Hodgkin lymphoma codes:  201, 201.0, 201.00, 201.01, 201.02, 201.03, 201.04, 201.05, 201.06, 201.07, 201.08, 201.1, 201.10, 201.11, 201.12, 201.13, 201.14, 201.15, 201.16, 201.17, 201.18, 201.2, 201.20, 201.21, 201.22, 201.23, 201.24, 201.25, 201.26, 201.27, 201.28, 201.4, 201.40, 201.41, 201.42, 201.43, 201.44, 201.45, 201.46, 201.47, 201.48, 201.5, 201.50, 201.51, 201.52, 201.53, 201.54, 201.55, 201.56, 201.57, 201.58, 201.6, 201.60, 201.61, 201.62, 201.63, 201.64, 201.65, 201.66, 201.67, 201.68, 201.7, 201.70, 201.71, 201.72, 201.73, 201.74, 201.75, 201.76, 201.77, 201.78, 201.9, 201.90, 201.91, 201.92, 201.93, 201.94, 201.95, 201.96, 201.97, 201.98 |
